# Mammographic density and breast cancer in women from high risk families

**DOI:** 10.1186/s13058-015-0604-1

**Published:** 2015-07-11

**Authors:** Teresa Ramón y Cajal, Isabel Chirivella, Josefa Miranda, Alexandre Teule, Ángel Izquierdo, Judith Balmaña, Ana Beatriz Sánchez-Heras, Gemma Llort, David Fisas, Virginia Lope, Elena Hernández-Agudo, María José Juan-Fita, Isabel Tena, Luis Robles, Carmen Guillén-Ponce, Pedro Pérez-Segura, Mari Sol Luque-Molina, Susana Hernando-Polo, Mónica Salinas, Joan Brunet, María Dolores Salas-Trejo, Agustí Barnadas, Marina Pollán

**Affiliations:** Medical Oncology Department, Hospital Santa Creu I Sant Pau, Barcelona, Spain; Medical Oncology Department, Hospital Clinico Universitario de Valencia, Valencia, Spain; Foundation General Directorate Public Health and Foundation for the Promotion of Health and Biomedical Research in the Valencian Region, FISABIO – Public Health, Valencia, Spain; Hereditary Cancer Program, Catalan Institue of Oncology-IDIBELL, Barcelona, Spain; Hereditary Cancer Program, Catalan Institute of Oncology-IDIBGI, Girona, Spain; Medical Oncology Deartment, Hospital Vall Hebron/Vall Hebron Institute of Oncology, Barcelona, Spain; Medical Oncology Department, Hospital Universitario de Elche, Elche, Spain; Genetic Counseling Unit, Corporació Sanitaria Parc tauli, Consorci Sanitari de Terrassa, Terrasa, Spain; National Center for Epidemiology, Carlos III Institute of Health, Monforte de Lemos 5, 28029 Madrid, Spain; Consortium for Biomedical Research in Epidemiology and Public Health (CIBERESP), Carlos III Institute of Health, Madrid, Spain; Consortium Cancer Epidemiology Research Group, Oncology and Hematology Area, IIS Puerta de Hierro (IDIPHIM), Madrid, Spain; Breast Cancer Unit, Clinical Research Programme, Spanish National Cancer Center (CNIO), Madrid, Spain; Medical Oncology Department, Foundation of the Valencian Oncologic Institute, Valencia, Spain; Medical Oncology Department, Hospital Provincial de Castellón, Castellón, Spain; Medical Oncology Department, Hospital 12 de Octubre, Madrid, Spain; Medical Oncology Department, Hospital Universitario Ramón y Cajal, IRYCIS, Madrid, Spain; Medical Oncology Department, Hospital Clínico San Carlos, Madrid, Spain; Gynecological Department, Hospital Gregorio Marañón, Madrid, Spain; Medical Department, Hospital Fundación Alcorcón, Alcorcón, Spain; Medical Sciences Department, School of Medicine, University of Girona, Girona, Spain

## Abstract

**Introduction:**

Mammographic density (MD) is one of the strongest determinants of sporadic breast cancer (BC). In this study, we compared MD in *BRCA1/2* mutation carriers and non-carriers from *BRCA1/2* mutation-positive families and investigated the association between MD and BC among *BRCA1/2* mutation carriers per type of mutation and tumor subtype.

**Methods:**

The study was carried out in 1039 female members of *BRCA1* and *BRCA2* mutation-positive families followed at 16 Spanish Genetic Counseling Units. Participants’ density was scored retrospectively from available mammograms by a single blinded radiologist using a 5-category scale (<10 %, 10-25 %, 25-50 %, 50-75 %, >75 %). In BC cases, we selected mammograms taken prior to diagnosis or from the contralateral breast, whereas, in non-cases, the last screening mammogram was evaluated. MD distribution in carriers and non-carriers was compared using ordinal logistic models, and the association between MD and BC in *BRCA1/2* mutation carriers was studied using logistic regression. Huber-White robust estimators of variance were used to take into account correlations between family members. A similar multinomial model was used to explore this association by BC subtype.

**Results:**

We identified and scored mammograms from 341 *BRCA1*, 350 *BRCA2* mutation carriers and 229 non-carriers. Compared to non-carriers, MD was significantly lower among *BRCA2* mutation carriers (odds ratio (OR) =0.71; *P-*value=0.04), but not among *BRCA1* carriers (OR=0.84; *P-*value=0.33). MD was associated with subsequent development BC (OR per category of MD=1.45; 95 % confidence interval=1.18-1.78, *P-*value<0.001), with no significant differences between *BRCA1* and *BRCA2* mutation carriers (*P-*value=0.48). Finally, no statistically significant differences were observed in the association of MD with specific BC subtypes.

**Conclusions:**

Our study, the largest to date on this issue, confirms that MD is an independent risk factor for all BC subtypes in either *BRCA1* and *BRCA2* mutation carriers, and should be considered a phenotype risk marker in this context.

## Introduction

Mammographic density (MD) is one of the strongest determinants of breast cancer (BC) in most ethnic groups. In general population studies, higher MD is consistently associated with a higher BC risk [1]. Individual variation of MD does not only depend on well-established epidemiologic factors, such as age, parity, menopausal status, external hormonal manipulation or body mass index (BMI), but based on family studies [2–7], it also depends on the as yet unknown genetic background.

Knowledge of the genetic basis of MD could be a major milestone in BC risk prediction and prevention. Previous candidate gene analysis and linkage studies have shown either inconclusive or discordant results to date. In contrast, genome-wide association studies have started to identify some genetic variants that could explain part of the variation in MD [8]. Furthermore, some of the well-established BC susceptibility variants are also associated with MD variability [9, 10].

Germline mutations in *BRCA1* and *BRCA2* genes are the most frequent cause of strong genetic predisposition to breast and ovarian cancer. In the last decades, several studies have evaluated whether the mutational status of both genes is associated with differences in MD compared to the general population. Therefore, whereas earlier studies with limited numbers of patients [11–16] had conflicting results, two larger and well-conducted studies compared MD in either affected or healthy *BRCA1/2* mutation carriers [17] and in mutation carriers versus women with low to average risk [18]. In one of them, authors proved MD to be an independent risk factor for BC in carriers [17]. In contrast, findings from a recent study did not reveal any association between baseline MD and subsequent risk of BC among carriers [19].

In this paper, we aimed to compare MD in Spanish *BRCA1/2* mutation carriers and non-carriers and assess the potential impact of MD on subsequent BC risk among carriers according to the mutated gene and pathologic subtype.

## Methods

A total of 1,039 women were enrolled from 509 breast and ovarian cancer families who had been counseled and tested for mutations in *BRCA1* and *BRCA2* genes, from January 1993 to December 2012, and followed up at 16 Cancer Genetic Units in Spain. The study was approved by national and regional Ethics Committees (see Acknowledgments for details) and conducted in compliance with the Helsinki Declaration. All women were older than 18 years and gave written informed consent for the study.

Eligible participants were women from mutation-positive families, including *BRCA1/2* mutation carriers and true mutation-negative women. Women diagnosed with BC were considered cases, whereas those individuals with no personal history of BC were regarded as non-cases or controls.

Structured questionnaires collecting baseline information were administered by investigators during either the high-risk breast screening or the post-test visit. Apart from demographic characteristics, data for other covariates were collected, such as the genetic condition, age, weight and height, reproductive history, menopausal status, the use of exogenous hormonal supplements and history of risk-reducing surgery up to the time of the study mammogram. Controls’ weight and height were measured by the investigator at the enrollment visit. Tumor subtype, weight and height at the time of the scored mammogram were extracted from the medical charts of BC patients.

Mammograms were either obtained from the radiological chart or provided by the participants. Due to the retrospective design of our study, we considered density measures from analog and digital mammographic films. For cancer patients, we aimed to collect the earliest mammogram obtained before diagnosis (within a 10-year period and identical menopausal status) and excluded the films from the affected breast. When unavailable, we used the mammogram of the unaffected contralateral breast taken at the time of cancer diagnosis. To make the age of controls at the time of the mammogram more comparable with those of women with BC, we attempted to obtain the latest available mammogram. For each participant, films from craniocaudal and mediolateral oblique views were requested. There were 119 women who either did not have a suitable mammogram (110) or the quality of the film was insufficient (9), rendering a total of 920 individuals for whom the mammogram was available and MD reading was feasible. There were 691 women carrying a deleterious mutation in the *BRCA1* or *BRCA2* (341 women with *BRCA1* and 350 with *BRCA2*) genes and 229 women were relatives without a mutation.

Breast density was visually assessed by one experienced radiologist, with high intra-observer concordance [20]. The radiologist was blinded to the diagnosis, the carrier status and the referral centre of the mammogram. MD was classified using the Boyd semiquantitative scale into 6 categories, namely 0 %, <10 %, 10−25 %, 25−50 %, 50−75 % and >75 %. The radiologist provided a single reading per participant, considering the craniocaudal and mediolateral oblique views in both breasts when available. For those breast cancer cases without previous mammography, the two views of the contralateral breast were read.

The association between MD and the carrier status as well as other variables of interest was assessed in healthy women using an ordinal logistic regression model. This procedure results from fitting k-1 logistic regression models, dichotomizing MD into two groups, i.e., high versus low MD, using all possible cutoffs. This model, also known as the proportional odds model, assumes that odds ratios (ORs) remain constant irrespective of the cutoff chosen, but allows the intercepts to differ. The following variables were included as possible confounders: age at mammogram, BMI, menopausal status (premenopause/natural menopause/surgical menopause), parity (nulliparous/parous), type of image (analog/digital) and carrier status (non-carrier, *BRCA1*, *BRCA2*). The correlation between members of the same family was taken into account using Huber-White robust estimators of variance, considering individuals clustered inside families [21]. The Brant test was used to verify the proportional odds assumption.

The association between MD and subsequent BC development was evaluated using a logistic regression model, adjusted for age at mammogram, menopausal status (premenopause/natural menopause/surgical menopause), BMI, parity, age at first live birth, use of hormonal replacement therapy (HRT) (never/ever), type of image and time elapsed from mammogram to either BC diagnosis (cases) or end of follow up (non-cases). The consistency of the association between MD and BC was explored performing subgroup analysis in models including MD as a continuous variable and an interaction term between MD and each of the explanatory variables already mentioned. Finally, possible differences in the effect of MD per BC subtype were assessed using a multinomial logistic model, adjusted for the same confounders and considering the following subtypes: 1) hormonal receptor positive (estrogen receptor (ER) or progesterone receptor (PR)) and human epidermal growth factor receptor 2 (HER2) negative, 2) HER2-positive, 3) triple-negative tumors, and 4) unknown subtype. Again, Huber-White robust estimators of variance were used in all instances to take into account the correlation between relatives [21]. Sensitivity analyses were carried out considering only those cases with mammograms taken 6 months or more before BC diagnosis. All statistical analyses were performed by using the STATA version 12.0 software program (Stata Corp, College Station, TX, USA).

## Results

### Baseline characteristics

Baseline characteristics of our population according to *BRCA1/2* mutation carrier status and development of breast cancer are shown in Table 1. Carriers were younger at the scored mammogram (mean age was 41 years, regardless of the mutated gene and disease condition) compared with non-carriers (45 years; *P* value <0.001). In addition, carriers were more likely to have ever used hormone preventive treatment (1.4 % vs 0.4 %; *P* value = 0.06) and have undergone surgical menopause at the time of the selected mammogram (10.3 % vs 1.7 %; *P* value <0.001). There were 31 women, 21 *BRCA1* and 10 *BRCA2* mutation carriers, with a previous diagnosis of ovarian cancer, and four of them (2 *BRCA1* and 2 *BRCA2* mutation carriers) subsequently developed a breast tumor.

**Table 1:** Characteristics of the women from *BRCA1/2* families included in the study

	*BRCA1/2* mutation carriers			Relatives without mutation		
Variable	Without BC	BC patients	*P* value^a^	Without BC	BC patients	*P* value^a^
	N = 443	N = 248		N = 219	N = 10	
Affected gene			0.183			.
*BRCA1*	227 (51.2 %)	114 (46.0 %)				
*BRCA2*	216 (48.8 %)	134 (54.0 %)				
Age at mammography			0.909			0.907
Mean (SD)	41.6 (11.5)	41.5 (10.2)		45.6 (11.8)	45.2 (11.2)	
Body mass index			0.030			0.948
<18.5	6 (1.4 %)	7 (2.8 %)		11 (5.0 %)	0 (0.0 %)	
18.5−24.9	259 (58.5 %)	151 (60.9 %)		120 (54.8 %)	7 (70.0 %)	
25−29.9	110 (24.8 %)	69 (27.8 %)		56 (25.6 %)	2 (20.0 %)	
30−34.9	49 (11.1 %)	19 (7.7 %)		28 (12.8 %)	1 (10.0 %)	
>=35	19 (4.3 %)	2 (0.8 %)		4 (1.8 %)	0 (0.0 %)	
Unknown	0 (0.0 %)	0 (0.0 %)		11 (5.0 %)	0 (0.0 %)	
Menopausal status^b^			0.239			0.576
Premenopausal	310 (70.0 %)	184 (74.2 %)		147 (67.1 %)	7 (70.0 %)	
Postmenopausal	133 (30.0 %)	64 (25.8 %)		72 (32.9 %)	3 (30.0 %)	
Type of menopause^b,c^			<0.001			0.154
Natural	73 (16.5 %)	53 (21.4 %)		69 (95.8 %)	2 (66.7 %)	
Surgery	60 (13.5 %)	11 (4.4 %)		3 (4.2 %)	1 (33.3 %)	
Time since menopause^b,c^			0.100			1.000
<=5 years	31 (7.0 %)	19 (7.7 %)		27 (12.3 %)	1 (10.0 %)	
6−10 years	34 (7.7 %)	8 (3.2 %)		17 (7.8 %)	1 (10.0 %)	
>10 years	63 (14.2 %)	35 (14.1 %)		28 (12.8 %)	1 (10.0 %)	
Unknown	5 (1.1 %)	2 (0.8 %)		0 (0.0 %)	0 (0.0 %)	
Nulliparous			0.151			0.264
No	302 (68.2 %)	182 (73.4 %)		167 (76.3 %)	6 (60.0 %)	
Yes	141 (31.8 %)	66 (26.6 %)		52 (23.7 %)	4 (40.0 %)	
Age at first birth, years^d^			0.110			0.464
>30	75 (16.9 %)	34 (13.7 %)		46 (27.5 %)	0 (0.0 %)	
26−30	111 (25.1 %)	64 (25.8 %)		60 (35.9 %)	3 (50.0 %)	
21−25	98 (22.1 %)	77 (31.0 %)		53 (31.7 %)	3 (50.0 %)	
<=20	18 (4.1 %)	7 (2.8 %)		8 (4.8 %)	0 (0.0 %)	
Oral contraceptives			0.487			0.508
No	156 (35.2 %)	81 (32.7 %)		58 (26.5 %)	4 (40.0 %)	
Yes	239 (54.0 %)	140 (56.5 %)		130 (59.4 %)	6 (60.0 %)	
Unknown	48 (10.8 %)	27 (10.9 %)		31 (14.2 %)	0 (0.0 %)	
Use of hormone replacement therapy^b^			0.613			0.246
No	431 (97.3 %)	238 (96.0 %)		205 (93.6 %)	9 (90.0 %)	
Current use	3 (0.7 %)	3 (1.2 %)		5 (2.3 %)	1 (10.0 %)	
Past use	9 (2.0 %)	7 (2.8 %)		9 (4.1 %)	0 (0.0 %)	
Use of hormonal preventive treatment^b^			0.349			
No	438 (98.9 %)	243 (98.0 %)		219 (99.5 %)	10 (100 %)	
Current use	5 (1.1 %)	5 (2.0 %)		1 (0.5 %)	0 (0.0 %)	
Type of mammogram			<0.001			0.020
Analog	132 (29.8 %)	208 (83.9 %)		62 (28.3 %)	6 (60.0 %)	
Digital	311 (70.2 %)	40 (16.1 %)		157 (71.7 %)	4 (40.0 %)	
Number of projections			<0.001			0.069
One	9 (2.0 %)	6 (2.4 %)				
Two	34 (7.7 %)	74 (29.8 %)		11 (4.7 %)	3 (25.0 %)	
Three	2 (0.5 %)	3 (1.2 %)		1 (0.4 %)	0 (0.0 %)	
Four	398 (89.8 %)	165 (66.5 %)		223 (94.9 %)	9 (75.0 %)	
Mammographic density			0.001			0.316
0 %	6 (1.4 %)	0 (0.0 %)		3 (1.4 %)	1 (10.0 %)	
<=10 %	99 (22.3 %)	35 (14.1 %)		44 (20.1 %)	1 (10.0 %)	
11−25 %	89 (20.1 %)	47 (19.0 %)		44 (20.1 %)	2 (20.0 %)	
26−50 %	151 (34.1 %)	85 (34.3 %)		72 (32.9 %)	3 (30.0 %)	
51−75 %	72 (16.3 %)	47 (19.0 %)		37 (16.9 %)	3 (30.0 %)	
>75 %	26 (5.9 %)	34 (13.7 %)		19 (8.7 %)	0 (0.0 %)	
Time of follow up^e^			<0.001			0.924
<=1 year	207 (46.7 %)	162 (65.3 %)		103 (47.0 %)	4 (40.0 %)	
1−2 years	82 (18.5 %)	30 (12.1 %)		39 (17.8 %)	2 (20.0 %)	
2−5 years	94 (21.2 %)	30 (12.1 %)		48 (21.9 %)	3 (30.0 %)	
5−10 years	46 (10.4 %)	24 (9.7 %)		22 (10.0 %)	1 (10.0 %)	
>10 years	14 (3.2 %)	2 (0.8 %)		7 (3.2 %)	0 (0.0 %)	
Prophylatic mastectomy			0.003			
No	428 (96.6 %)	248 (100.0 %)				
Yes	15 (3.4 %)	0 (0.0 %)				
Breast cancer features						
Histology						.
Ductal in situ		15 (6.0 %)			3 (30.0 %)	
Ductal		187 (75.4 %)			6 (60.0 %)	
Lobular		12 (4.8 %)			1 (10.0 %)	
Medular		14 (5.6 %)			0 (0.0 %)	
Others		18 (7.3 %)			0 (0.0 %)	
Unknown		2 (0.8 %)			0 (0.0 %)	
Type of breast cancer						
ER/PR+&HER2−		99 (39.9 %)			6 (60.0 %)	
HER2+		23 (9.3 %)			1 (10.0 %)	
Triple-negative		74 (29.8 %)			1 (10.0 %)	
Unknown		52 (21.0 %)			2 (20.0 %)	

^a^Comparison between women who did and did not develop breast cancer. ^b^At time of mammography. ^c^Only postmenopausal women at time of mammography. ^d^Only parous women. ^e^Time elapsed from mammographic exploration to breast cancer diagnosis (cases) or last contact (controls). *BC* breast cancer, *ER* estrogen receptor, *PR* progesterone receptor, *HER2* human epidermal growth factor receptor 2

Among 691 *BRCA1/2* mutation carriers, 233 women developed invasive breast cancer (111 and 122 with *BRCA1* and *BRCA2* mutations, respectively), and 15 patients had ductal carcinoma in situ (3 *BRCA1* and 12 *BRCA2* mutation carriers, respectively), whereas 7 women had invasive breast cancer and the other 3 women developed ductal carcinoma in situ, among 229 non-carriers. Regarding pathologic subtypes, 54 cases, 2 of them among non-carriers, could not be classified due to lack of information on hormone receptor expression (11 cases) or HER2 status (54 cases). As expected, *BRCA2* mutation carriers were more likely to have hormone receptor positive tumors and no expression of HER2 (71 % versus 26 %; *P* value <0.001), whereas *BRCA1* mutation carriers had a higher proportion of triple-negative tumors (66 % versus 14 %; *P* value <0.001).

When we looked specifically at *BRCA1/2* mutation carriers, breast cancer patients had a lower BMI compared to controls, with 92 % of cases versus 85 % of controls having BMI <30 (*P* value = 0.03) (Table 1). No differences were seen in the age at the time of scored mammogram, but analog mammography was more frequent among cases (84 % versus 30 %). Finally, MD was higher in affected carriers, with 33 % of them in the two highest categories (MD >50 %) as opposed to 22 % of controls (*P* value = 0.001). Menopausal status, parity, age at first birth, oral contraceptives and hormonal replacement therapy were not significantly different between both groups. As expected, surgical menopause was more frequent among controls (14 % versus 4 %; *P* value <0.001).

### Association of mammographic density with other variables

The association of all categories of mammographic density and explanatory variables is shown in Table 2. Given the small number of women with 0 % MD, the two first categories were combined in subsequent analyses. In all instances, ORs were adjusted for age at mammogram, BMI, menopausal status (premenopause/natural menopause/surgical menopause), type of mammogram, parity, carrier status and subsequent development of BC. We did not find a significant association between carrying a *BRCA1/2* mutation and a lower MD (OR = 0.77; 95 % CI = 0.57, 1.05). However, when *BRCA1* and *BRCA2* mutation carriers were considered separately, *BRCA2* mutation carriers had a statistically significant lower MD (OR = 0.71; 95 % CI = 0.51, 0.98: *P* value = 0.04), but no differences were observed for *BRCA1* mutation carriers (*P* value = 0.332). Most risk factors considered, including age at mammography, BMI, menopausal status and parity were, as expected, significantly associated with MD. Moreover, MD was higher among women who subsequently developed a breast tumor (OR = 1.69; 95 % CI = 1.24, 2.31; *P* value = 0.001), Finally, neither age at first birth nor hormonal interventions such as HRT or oral contraceptives (OC) significantly modified MD in our study.

**Table 2:** Association of mammographic density with carrier status and other characteristics in healthy female mutation carriers (controls) from *BRCA1/BRCA2* families

		Categories of mammographic density							
Variable	Number	<=10 %	11−25 %	26−50 %	51−75 %	>75 %	Odds ratio^a^	95 % CI^a^	*P* value
Carrier status									
Non-carrier	219	21 %	20 %	33 %	17 %	9 %	1.00		
*BRCA1* or *BRCA2*	443	24 %	20 %	34 %	16 %	6 %	0.75	0.54, 1.04	0.085
*BRCA1*	227	22 %	25 %	29 %	19 %	6 %	0.84	0.58, 1.23	0.376
*BRCA2*	216	26 %	15 %	39 %	14 %	6 %	0.67	0.47, 0.96	0.028
Age at mammography, years									
<35	188	10 %	14 %	36 %	27 %	13 %	1.00		
35−44	199	17 %	19 %	39 %	17 %	8 %	0.94	0.54, 1.62	0.826
45−54	170	29 %	25 %	31 %	12 %	3 %	0.79	0.33, 1.90	0.603
>=55	105	49 %	25 %	24 %	3 %	0 %	0.57	0.15, 2.20	0.415
Linear trend (per year)							0.97	0.95, 0.99	<0.001
Body mass index									
<25	396	10 %	17 %	39 %	24 %	10 %	1.00		
25.0−29.9	166	36 %	26 %	28 %	7 %	3 %	0.28	0.19, 0.40	<0.001
>=30	100	51 %	24 %	23 %	2 %	0 %	0.16	0.10, 0.25	<0.001
Linear trend (per unit)							0.83	0.80, 0.86	<0.001
Menopausal status & type of menopause									
Premenopausal	457	15 %	17 %	37 %	21 %	9 %	1.00		
Natural menopause	142	41 %	24 %	28 %	6 %	1 %	0.72	0.45, 1.16	0.179
Surgical menopause	63	38 %	35 %	21 %	6 %	0 %	0.56	0.33, 0.96	0.034
Nulliparous									
No	460	29 %	23 %	33 %	13 %	2 %	1.00		
Yes	202	9 %	14 %	35 %	24 %	17 %	1.87	1.28, 2.73	0.001
Age at first birth, years (only parous women)									
>30	131	25 %	17 %	35 %	17 %	6 %	1.00		
25−29	170	25 %	25 %	34 %	15 %	1 %	1.17	0.72, 1.88	0.526
20−24	151	35 %	24 %	30 %	9 %	2 %	1.10	0.67, 1.82	0.706
<20	26	38 %	23 %	27 %	12 %	0 %	0.72	0.31, 1.66	0.436
Linear trend (per year)							1.00	0.97, 1.05	0.745
Hormonal replacement therapy									
No	636	23 %	20 %	34 %	17 %	7 %	1.00		
Yes	26	23 %	27 %	27 %	15 %	8 %	1.71	0.87, 3.38	0.120
Hormonal preventive therapy									
No	656	23 %	20 %	34 %	17 %	7 %	1.00		
Yes	6	33 %	17 %	50 %	0 %	0 %	0.67	0.25, 1.82	0.434
Oral contraception									
No	214	22 %	18 %	34 %	19 %	7 %	1.00		
Yes	369	22 %	21 %	33 %	17 %	7 %	0.93	0.67, 1.27	0.637
Type of image									
Analog	194	26 %	21 %	34 %	13 %	6 %	1.00		
Digital	468	22 %	20 %	34 %	18 %	7 %	1.09	0.79, 1.50	0.588
Number of projections									
One or two	53	15 %	17 %	40 %	21 %	8 %	1.00		
Three or Four	609	24 %	20 %	33 %	16 %	7 %	1.17	0.70, 1.94	0.544

^a^Adjusted for age at mammogram, body mass index, menopause (premenopausal, natural menopause, surgical menopause), type of mammogram, parity (nulliparous versus parous ), carrier status (non-carrier, *BRCA1*, *BRCA2*) and subsequent development of breast cancer (no, yes)

### Effect of mammographic density in development of breast cancer in *BRCA1/2* mutation carriers

**Fig. 1:**
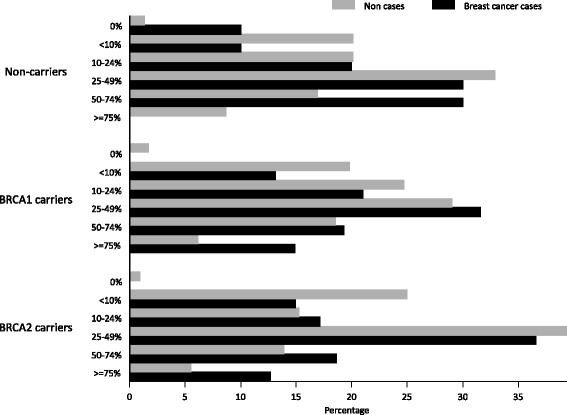
Distribution of mammographic density in breast cancer cases and non-cases in the following groups: non-carriers, *BRCA1* mutation carriers and *BRCA2* mutation carriers

The distribution of MD in BC cases and non-cases for women who were non-carriers and *BRCA1* and *BRCA2* mutation-carriers is presented in Fig. 1. Cases tended to be more frequently classified in the higher MD categories. Given the limited number of BC cases among the non-carriers group, we did not evaluate the potential different effect of MD on cancer risk in non-carriers. Table 3 shows the association between MD and subsequent development of BC among *BRCA1/2* mutation carriers. After adjusting for age at mammogram, BMI, menopause (premenopause/natural menopause/surgical menopause), parity, age at first birth, HRT, type of mammogram and time of follow up, there was a clear association between MD and subsequent BC development among *BRCA1/2* mutation carriers, with a statistically significant dose-response trend (*P* value <0.001). The OR in women with density 50−75 % relative to women with density ≤10 % was 3.24 (95 % CI = 1.43, 7.35; *P* value = 0.005), while women in the highest MD category, namely >75 %, had an OR of 4.34 (95 % CI = 1.71, 11.1; *P* value = 0.002). The association between MD and BC tended to be stronger among *BRCA2* mutation carriers (OR per increase in one category of MD of 1.60 in *BRCA2* and of 1.37 in *BRCA1* carriers), but these differences were not statistically significant (*P* value for heterogeneity = 0.449). Sensitivity analysis including only the cases with a mammogram obtained at least 6 months before cancer diagnosis had comparable results (see the central columns in Table 3). Finally, given that most mammograms from BC cases were analog, the last columns present the association between MD and BC using only analog images (Table 3). ORs tended to be stronger in this case, particularly for *BRCA2* mutation carriers. The analysis of digital images was hampered by the small number of BC cases available (40 cases: 6 MD <=10 %, 6 MD = 11−25 %, 17 MD = 26−50 %, 9 MD = 51−75 % and 2 MD >75 %) and no association between MD and BC was observed (ORs of 1.23, 1.94, 1.88 and 1.22, respectively; none of the ORs were statistically significant).

**Table 3:** Association of mammographic density with subsequent breast cancer in *BRCA1/2* mutation carriers

		All cases included				Excluding cases without a mammogram taken at least 6 months before diagnosis				Including only analog images				
Mammographic density	Non-cases	BC cases	OR^a^	95 % CI^a^	*P* value^a^	BC cases^a^	OR^a^	95 % CI^a^	*P* value^a^	Non-cases	BC cases	OR^b^	95 % CI^b^	*P* value^b^
*BRCA1*+*BRCA2*														
<=10 %	105	35	1.00			12	1.00			36	29	1.00		
1125 %	89	47	1.51	0.74, 3.06	0.255	18	1.76	0.70, 4.41	0.228	29	41	1.74	0.72, 4.19	0.216
26−50 %	151	85	1.85	0.90, 3.82	0.095	39	2.26	0.94, 5.44	0.070	45	68	1.77	0.75, 4.15	0.191
51−75 %	72	47	3.24	1.43, 7.35	0.005	21	3.67	1.37, 9.80	0.010	15	38	4.88	1.72, 13.9	0.003
>75 %	26	34	4.34	1.71, 11.1	0.002	17	8.94	2.86, 28.0	<0.001	7	32	9.45	2.73, 32.7	<0.001
Linear trend	443	248	1.45	1.18, 1.78	<0.001	107	1.64	1.28, 2.09	<0.001	132	208	1.68	1.31, 2.15	<0.001
*BRCA1*														
<=10 %	49	15	1.00			3	1.00			15	14	1.00		
11−25 %	56	24	2.18	0.85, 5.58	0.103	8	2.71	0.63, 11.7	0.183	15	20	1.78	0.50, 6.48	0.370
26−50 %	66	36	1.90	0.78, 4.67	0.160	14	3.06	0.73, 12.9	0.127	23	30	1.15	0.39, 3.39	0.798
51−75 %	42	22	2.53	0.89, 7.18	0.082	10	4.22	0.93, 19.2	0.063	9	21	3.68	0.75, 18.1	0.109
>75 %	14	17	5.42	1.79, 16.4	0.003	9	12.0	2.20, 65.1	0.004	3	16	7.49	1.57, 35.7	0.012
Linear trend	227	114	1.37	1.06, 1.76	0.015	44	1.65	1.17, 2.32	0.004	65	101	1.51	1.08, 2.12	0.017
*BRCA2*														
<=10 %	56	20	1.00			9	1.00			21	15	1.00		
11−25 %	33	23	1.29	0.45, 3.68	0.639	10	1.70	0.47, 6.19	0.419	14	21	2.21	0.76, 6.46	0.146
26−50 %	85	49	2.03	0.65, 6.34	0.222	25	3.36	0.89, 12.7	0.074	22	38	3.85	1.06, 13.9	0.040
51−75 %	30	25	5.37	1.35, 21.4	0.017	11	7.38	1.26, 43.1	0.027	6	17	11.2	1.82, 68.9	0.009
>75 %	12	17	4.82	1.10, 21.1	0.037	8	13.4	2.33, 77.3	0.004	4	16	18.8	3.06, 114.9	0.002
Linear trend	216	134	1.60	1.13, 2.28	0.008	63	1.95	1.30, 2.91	0.001	67	107	2.11	1.40, 3.16	<0.001

^a^Odds ratio (*OR*), 95 % CI and *P* values adjusted for age at mammogram, menopausal status premenopausal, natural menopause, surgical menopause), body mass index, parity (nulliparous, parous), age at first live birth, use of hormonal replacement treatment (yes, no), type of image (analog, digital) and time elapsed from mammogram to breast cancer diagnosis (cases) or end of follow up (non-cases). ^b^OR, 95 % CI and *P* values adjusted for the above-mentioned variables except type of image

**Fig. 2:**
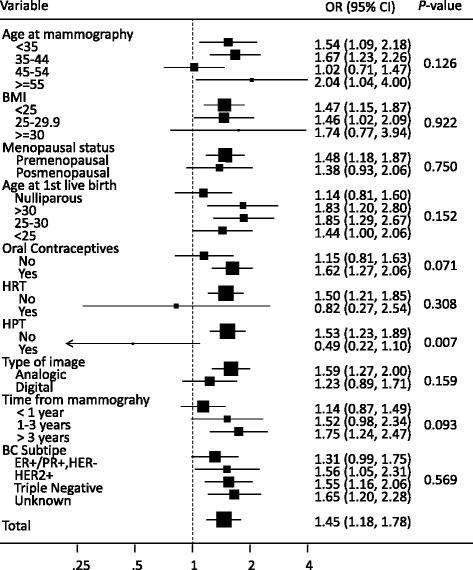
Association between mammographic density (MD) and subsequent breast cancer (BC) among *BRCA1* or *BRCA2* mutation carriers: analysis stratified by other explanatory variables. Odds ratios (*OR*) and 95 % CI correspond to an increase in one category of MD. Estimators adjusted for age at mammogram, menopausal status, body mass index (*BMI*), parity, age at first live birth, use of hormonal replacement therapy (*HRT*), type of image and time elapsed from mammogram to either breast cancer diagnosis (cases) or end of follow up (non-cases). *P* value reflects the statistical significance of the interaction term between MD and the corresponding variable in all cases except for BC subtype, for which the *P* value is for the test of heterogeneity of the MD effect according to the type of tumor. *HPT* hormone preventative treatment, *ER* estrogen receptor, *PR* progesterone receptor, *HER2* human epidermal growth factor receptor 2

The association between MD (as an ordinal variable) and BC per category of the other explanatory variables among mutation carriers is presented in Fig. 2. The OR represents the risk excess associated with an increase in one category of MD. In general, a consistent effect of MD on BC risk was seen within all subgroup analyses. Even though the association seemed to be less marked among women older than 45 years at the time of mammography, those who were nulliparous, and those who had not taken OC, none of these differences were statistically significant. The smaller number of women who reported the use of HRT was too small to reach conclusions in this group. The association with the type of image was stronger for analog mammograms, but most cases had analog mammograms, whereas the opposite was true for non-cases. Finally, MD increased the risk of all pathologic subtypes BC subtypes in a similar way (*P* value for heterogeneity = 0.638).

## Discussion

In this case-control study of women from high-risk breast cancer families, we confirmed that breast density was an independent risk factor for BC among *BRCA1* and *BRCA2* mutation carriers. Compared to women with breast density <10 %, those with densities >50 % had at least threefold increased risk of developing a breast tumor. The association between MD and the risk of subsequent BC seemed to be stronger for *BRCA2* than for *BRCA1* mutation carriers, but the sample size was insufficient to detect statistical differences between them. Our results also confirmed an association between MD and all pathologic BC subgroups. Finally, we found that mutation carriers had lower MD estimations compared to non-carriers and this difference remained significant among *BRCA2* mutation carriers.

The role of MD as a risk predictor for BC has relevant implications in screening and preventive interventions, particularly in highly motivated women with a family history of the disease. Our results were consistent with early evidence for the association between BC risk and MD among women from high-risk families [6, 7] and later findings on *BRCA1* and *BRCA2* mutation carriers [17, 18], assessed by computerized methods for obtaining quantitative measures of MD. Initial case-control studies evaluating the association between family history, MD and subsequent risk of BC were completed by Martin et al. [7] in a sample of 2,322 subjects from three Canadian screening programs. In that population, MD explained 14 % of the association between family history and BC risk, being considered an intermediate marker for BC. Moreover, women with breast density >50 % had threefold higher risk of BC than those with <10 % density (95 % CI = 2.17, 4.15). Our OR for carriers with >50 % breast density are in agreement with those previously reported in our country among BC screening attendants, using the same semiquantitative visual scale [22]. In that study, the risk excess associated with higher MD was similar in women with and without a family history of BC, similar to the findings of the Nurses’ Health Study [23].

Raising the question of the potential impact of MD on women with a germline mutation in *BRCA1* and *BRCA2* genes, Mitchell et al. evaluated the association between MD and BC risk among 342 women from the EMBRACE study, 206 of whom were *BRCA* mutation carriers [17]. In spite of some methodological differences and a limited sample of participants, their results were comparable to ours. Assessing MD with a semi-automatic tool, the authors estimated the OR for BC among carriers with ≥50 % breast density to be 2.27 (95 % CI = 0.70, 7.39) compared with carriers who had <10 % breast density, while using a qualitative scale (Wolfe’s), the highest density group had a risk excess close to 3 (OR = 2.78) [17]. In contrast, a recent prospective nested case-control study investigating the same issue in a cohort of 462 BRCA mutation carriers enrolled in a BC screening program failed to confirm this association [19]. These negative results could be partially explained by facts such as the relatively small sample size and small number of cases, the high proportion of patients with a prior diagnosis and treatment of BC (19 %), the older mean age at the baseline mammogram, and the lack of information on BMI, one of the most important established confounding factors for MD. This confounding effect is particularly relevant when MD is assessed using computer-assisted tools, given the strong correlation between BMI and the non-dense area [24, 25]. Due to the potential impact of cancer treatment on MD, and similar to the EMBRACE study, we restricted our study to include only women with first diagnosis of BC as cases. Indeed, Passaperuma et al. did not provide information relative to adjuvant cancer therapy, which could have acted as a confounding factor [19]. In fact, based on the effect of tamoxifen in reducing MD, research has been lately focused on evaluating the potential use of MD variation as a target for identifying women who would benefit from this drug either as preventive or adjuvant therapy [26–28].

Despite initial conflicting results from a small series of patients, two later studies investigating the effect of mutation status on MD patterns have reported similar results. The first study was based on 505 retrospectively obtained mammographic readings from 342 women and found no significant difference in MD between carriers and non-carriers, either as a group or when mutated genes were considered separately [17]. In fact, the mean density was slightly lower in carriers than non-carriers. A second study compared MD between 143 healthy carriers and 119 women determined to be at low to average risk of BC [18]. In agreement with our results, the authors also found a marginally lower, albeit not significant, MD among carriers after adjustment for age and BMI. In our study, based on a larger sample size, mutation carriers had lower breast density compared to non-carriers, although this effect was only statistically significant for *BRCA2* mutation carriers.

As regards BC subtypes, previous studies have addressed the association between MD and BC in the general population with inconsistent results [22, 29–32]. Therefore, whereas a few studies have suggested that MD is mainly associated with hormone receptor positive tumors [32], others have reported a similar [22, 30] or event stronger association with triple-negative tumors, especially for women aged <55 years [29]. In addition, and similar to our results in *BRCA1/2* mutation carriers, a recent study in screening participants in Spain proved that high MD was associated with all histologic subtypes of BC [22].

Our study had several limitations, mostly related to its retrospective nature. Indeed, the heterogeneity of mammograms due to the use of multiple mammographers, the inclusion of both digital and analog images and differences in methodology, such as the subjective visual assessment of breast density and the use of semiquantitative assessments, limit the comparability of our results with other studies. Regarding the use of digital and analog mammograms, density tends to be lower when estimated via digital mammography and most images for BC cases were analog (84 % analog versus 16 % digital). Owing to this, we were able to show a clear effect of MD on BC risk in general and also restricting the analysis to cases and controls with analog images, but no effect was observed using digital mammograms, probably due to the small number of BCs in this category and the fact that only eight of them were classified in the extreme categories. Future studies are needed to confirm these results in mutation carriers studied with digital mammograms. Last, although the plan in original design of our study was to use healthy true negative *BRCA1/2* mutation carriers and phenocopies as controls for comparing the effect of MD on cancer risk between carriers and non-carriers, the smaller number of cases in this group did not allow us to accomplish this goal.

The strengths of our study include the fact that this is the largest cohort of women with a *BRCA1/2* mutation and MD data ever published. In spite of being a multicentric study, a single blinded radiologist, who is a highly reputed expert on mammographic reading, with a very high internal consistency [20], assessed all mammograms using a semiquantitative scale that has proved to be associated with subsequent development of BC [22]. Finally, restricting the analysis to BC patients with mammograms obtained 6 months before the diagnosis and the adjustment for all known confounders of MD provides compelling support for our results.

## Conclusions

This is the largest study to date to assess the influence of MD on BC risk and is concordant with previously published results in that MD is an independent risk factor among *BRCA1/2* mutation carriers. Even though MD was not increased among mutation carriers compared to non-carriers, high MD increased the risk of all pathologic subtypes of BC. Prospective studies are needed to clarify the impact of density variations on BC risk and provide a more pro-active prevention strategy for women harboring *BRCA1/2* mutations.

## Abbreviations

BC: breast cancer
BMI: body mass index
ER: estrogen receptor
HER2: human epidermal growth factor receptor 2
HPT: hormone preventative treatment
HRT: hormonal replacement treatment
MD: mammographic density
OC: oral contraceptives
OR: odds ratio
PR: progesterone receptor
